# Integrating Intestinal Ultrasound to Clinical Trials in Patients With Crohn’s Disease: Opportunities and Challenges

**DOI:** 10.1093/ibd/izaf196

**Published:** 2025-09-04

**Authors:** Vipul Jairath, Shashi Adsul, Mariangela Allocca, Silvio Danese, Marla C Dubinsky, Marcelo Freire de Oliveira, Christopher Ma, Torsten Kucharzik, Kerri L Novak, Remo Panaccione, Itzel Romo Bautista, Bruce E Sands, Stuart A Taylor, Rune Wilkens, Christian Maaser

**Affiliations:** Schulich School of Medicine & Dentistry, Western University, London, ON, Canada; Alimentiv, London, ON, Canada; Takeda, Cambridge, MA, United States; Gastroenterology and Endoscopy, IRCCS Hospital San Raffaele and University Vita-Salute San Raffaele, Milan, Italy; Gastroenterology and Endoscopy, IRCCS Hospital San Raffaele and University Vita-Salute San Raffaele, Milan, Italy; Dr. Henry D. Janowitz Division of Gastroenterology, Icahn School of Medicine at Mount Sinai, New York, NY, United States; Takeda, Cambridge, MA, United States; Division of Gastroenterology & Hepatology, Department of Medicine, Cumming School of Medicine, University of Calgary, AB, Canada; Klinikum Lüneburg, Lüneburg, Germany; Division of Gastroenterology & Hepatology, Department of Medicine, Cumming School of Medicine, University of Calgary, AB, Canada; Division of Gastroenterology & Hepatology, Department of Medicine, Cumming School of Medicine, University of Calgary, AB, Canada; Takeda, Cambridge, MA, United States; Dr. Henry D. Janowitz Division of Gastroenterology, Icahn School of Medicine at Mount Sinai, New York, NY, United States; Center for Medical Imaging, University College London, London, United Kingdom; Copenhagen University Hospital, Bispebjerg, Digestive Disease Center, Copenhagen, Denmark; Klinikum Lüneburg, Lüneburg, Germany

## Abstract

This narrative review summarizes the current knowledge on using intestinal ultrasonography (IUS) to evaluate disease activity in patients with Crohn’s disease (CD) and explores its potential role in clinical trials. Current trial endpoints and their limitations are discussed, highlighting the need for more patient-centric approaches, including increased use of magnetic resonance enterography (MRE) and IUS. Intestinal ultrasonography offers several advantages: it is noninvasive, requires no sedation, bowel preparation, or exposure to ionizing radiation, and enables real-time assessment of disease activity. It also demonstrates high sensitivity and specificity for detecting transmural inflammation and complications such as strictures, abscesses, and fistulas. Compared with cross-sectional imaging modalities like MRE and computed tomography, IUS is more patient-friendly, cost-effective, and suitable for point-of-care examination. However, challenges remain, including the lack of a universally accepted disease activity scoring system for MRE or IUS, despite the development and validation of several scoring tools. Key unmet needs include standardization of image acquisition and reporting, adequate training of healthcare professionals, improved access to equipment, and reimbursement pathways. Intestinal ultrasonography is increasingly being integrated into clinical trials to assess transmural inflammatory changes in CD, with IUS-based measures of transmural remission or response showing promise as potential endpoints. Although its advantages are clear, addressing these unmet needs is essential to broaden the adoption of IUS in both clinical trials and routine clinical practice.

## Background

Crohn’s disease (CD) is a chronic transmural inflammatory disease (IBD) of the intestine that involves periods of remission and relapses, and effective management strategies can help control symptoms, prevent bowel damage, and improve quality of life.^1^ Crohn’s disease is characterized by segmental transmural inflammation of the digestive tract, which can be complicated by strictures, fistulas, and abscesses.^2^ Knowledge of clinically relevant treatment targets is essential for improving and managing patients with CD.^3^ In addition, accurate, patient-friendly approaches to measuring these treatment targets are needed.

## Aim

This narrative review summarizes the current knowledge on using intestinal ultrasonography (IUS) to evaluate disease activity and treatment response in clinical trials and real-world clinical practice in patients with CD and also discusses the potential benefits and challenges of implementing IUS. Relevant studies were included from PubMed and Clinicaltrials.gov searches for CD and IUS or magnetic resonance enterography (MRE), and the results were supplemented by the authors’ knowledge of the disease area.

## Traditional endpoints in CD

### Clinical endpoints

The CD Activity Index (CDAI) has been historically used as an endpoint in CD clinical trials; however, concerns have been raised about the subjectivity of CDAI and the weak association between clinical symptoms measured by CDAI and the underlying inflammation and potential complications.^4^ The European Medicines Agency (EMA) and the US Food and Drug Administration (FDA) guidance on drug development for CD treatment recommends using coprimary endpoints in trials to ensure that treatments have a meaningful impact on the underlying bowel wall inflammation in addition to relieving signs and symptoms.^5^^,^^6^ Although the FDA recognizes the limitations of CDAI, they still recommend its use as a coprimary endpoint.^6^

### Biomarker endpoints

Although the FDA guidance does not mention biomarkers, the EMA encourages use of biomarkers of inflammation (C-reactive protein [CRP] and fecal calprotectin), while stating that there is insufficient evidence demonstrating that they can provide standalone evidence of inflammation.^5^^,^^6^ Instead, the EMA recommend use of biomarkers as secondary endpoints, highlighting biomarkers such as CRP and fecal calprotectin are surrogate markers of inflammation that should be con­sidered supplementary and not replace direct evaluation of inflammation.^5^

### Endoscopic endpoints

Both guidance documents recommend that endoscopic remission should be evaluated using a validated score, with the EMA recommending either the CD Endoscopic Index of Severity (CDEIS) or simple endoscopic score for CD (SES-CD), while the FDA recommends the SES-CD.^5^^,^^6^ The FDA recommends assessing endoscopic outcomes in CD clinical trials via blinded, standardized central reading to minimize bias.^6^ Endoscopy is the most widely used method for evaluating disease activity in CD; however, it has inherent limitations. Endoscopic scoring systems have been developed and validated for CD, including the CDEIS and the SES-CD. Still, the lack of validated thresholds for defining endoscopic remission and response remains a significant issue.^7^ Endoscopic procedures are invasive, reducing acceptance and compliance by patients. Intolerability limits repeatability, often leading to longer intervals between procedures.^8^ Endoscopic evaluation is limited to the mucosa and does not visualize the transmural inflammatory process, limiting the ability to fully characterize the disease burden, including the mesentery.^8^^,^^9^ Bowel strictures, a frequent CD complication, may preclude complete ileocolonoscopy (IC),^10^ thus limiting the feasibility of endoscopic monitoring for all cases. In addition, complications such as fistula and abscesses cannot be accurately detected by endoscopy. The proximal ileum is out of reach of the colonoscope; therefore, incomplete endoscopic evaluation can occur when inflammation is distributed proximal to the terminal ileum.^11^ Failure to comprehensively detect the extent of inflammation may result in delayed management of CD. The limitations of endoscopy have increased interest in cross-sectional imaging to evaluate disease activity and severity in CD.^12^

### Moving beyond traditional endpoints: the role of transmural imaging endpoints

The Selecting Therapeutic Targets in Inflammatory Bowel Disease (STRIDE)-II guidelines from the International Organization for the Study of Inflammatory Bowel Disease (IOIBD) also highlight the importance of treating underlying inflammation, with endoscopic remission, as measured by validated endoscopic scores included as a long-term treatment target, and transmural remission (also known as transmural healing^13^) recognized as an important adjunctive measure to assess the remission depth.^3^ Endpoints recommended by the STRIDE-II guidelines for patients with CD and outcomes recommended for CD disease-modification trials by the IOIBD SPIRIT consensus are shown in Table 1.^3^^,^^14^ Although transmural remission remains an aspirational target to date, evaluating the impact of treatment on transmural parameters is becoming increasingly relevant.^13^ One study that investigated transmural healing in patients with CD included 218 patients treated for 2 years with tumor necrosis factor antagonists, 31% (*n* = 68) had transmural healing, and 27% (*n* = 60) had mucosal healing.^15^ Patients with transmural healing had lower hospitalization and surgery rates at 1 year compared with those with mucosal healing alone, as well as longer times to clinical relapse, hospitalization, and surgery. Although transmural healing is an ambitious target, this study confirmed it is possible to achieve transmural healing, which is associated with significant improvements in clinical outcomes.^15^

**Table 1.** Endpoints recommended for clinical use in patients with CD from the STRIDE-II guidelines.

Endpoint	Treatment target	Definition (adults)	Definition (children)
Clinical response	Intermediate	A decrease of at least 50% in PRO2 (abdominal pain and stool frequency)	Decrease in PCDAI of at least 12.5 points and in wPCDAI at least 17.5 points
Clinical remission	Intermediate	PRO2 (abdominal pain ≤1 and stool frequency ≤3) or HBI <5	PCDAI (<10 points or <7.5 excluding the height item) or wPCDAI (<12.5 points)
Endoscopic remission	Long-term	SES-CD <3 points or absence of ulcerations (eg, SES-CD ulceration subscore = 0)	
Deep remission	Long-term	Absence of mucosal ulceration and a CDAI score <150	
CS-free remission	Intermediate	CS tapering during induction treatment	
Transmural remission	Not a target^a^		
Normalization of CRP and fecal calprotectin	Intermediate	CRP to values under the ULN and fecal calprotectin to 100–250 μg/g^b^	
Absence of disability and normalized HRQoL	Long-term		

a Should be used as an adjunct to endoscopic remission to represent a deeper level of healing.

b The cutoff value of fecal calprotectin is dependent on the desired outcome. Lower thresholds (eg, <100 μg/g) have been proposed for reflecting deep healing (both endoscopic and transmural healing) or histological healing, whereas higher values (eg, <250 μg/g) reflect less stringent outcomes.

Abbreviations: CRP, C-reactive protein; CS, corticosteroids; HBI, Harvey-Bradshaw index; HRQoL, health-related quality of life; PRO, patient-reported outcome; PRO2, patient-reported outcome based on stool frequency and rectal bleeding; SES-CD, Simple Endoscopic Score in Crohn’s disease; ULN, upper limit of normal; wPCDAI, weighted Pediatric Crohn’s Disease Activity Index.

## Imaging endpoints

Incorporation of transmural healing as a treatment target could improve patient care, not only through improvements in clinical outcomes, but also through the use of more patient-centric, noninvasive monitoring techniques such as MRE and IUS.

### Imaging charters—the regulatory approach to imaging endpoints

The FDA has released guidance to assist sponsors in optimizing imaging data quality obtained from clinical trials to support the approval of drugs and biological products.^16^ The guidance focuses on imaging acquisition, display, archiving, and interpretation process standards when imaging assesses a trial’s primary endpoint or component. If existing medical imaging practice standards need to be augmented to create trial-specific imaging process standards, these standards may be too lengthy to be included in the clinical protocol. In that case, the FDA encourages sponsors to develop an imaging charter that details the trial-specific imaging process standards.^16^ An *imaging charter*, consisting of a single document or a series of technical documents, should describe how potential sources of imaging bias and variability are controlled and how imaging process standards are implemented to a level appropriate to the trial design.^16^ The imaging charter should include modality-specific technical details, image acquisition protocols, data transfer plans, image submission guidelines, image interpretation, and image archiving procedures. The imaging charter should also be available to all clinical trial sites, prior to the trial commencing, to inform them of best practice.

### Magnetic resonance enterography

Magnetic resonance enterography is a cross-sectional imaging technique that can view the entirety of the bowel, particularly the small bowel. The accuracy for measuring disease activity is high due to its excellent soft tissue contrast.^17^^,^^18^ Magnetic resonance enterography has a lower sensitivity in colonic segments than endoscopy.^18^ In addition, although MRE can be used in perianal disease, perianal fistulae and abscesses can be missed when using a standard MRE protocol.^17^^,^^19^ It can measure treatment response and bowel damage via validated scores (such as Magnetic Resonance Index of Activity [MaRIA] and simplified MaRIA [sMaRIA]) and can visualize complications.^17^ Magnetic resonance enterography can also quantify small bowel motility, which can be a biomarker for disease activity, and has been linked to abdominal symptoms in patients, and is under investigation for stricture characterization.^20–22^ Advantages of MRE include good tissue contrast resolution, transmural and extramural inflammation assessment, and lack of ionizing radiation.^8^^,^^23^ Disadvantages of MRE include a relatively high cost and potentially cumbersome preparation (fasting for 4–6 hours before the procedure and ingestion of a significant volume of oral contrast). Other disadvantages include lower availability and access vs other modalities such as computed tomography, extended procedure times (at least 20–35 minutes), a requirement for patients to lie still and to undertake multiple breath holds during the procedure to avoid motion artifacts, and the potential need for sedation in patients with claustrophobia.^8^^,^^23–25^ In addition, there is a relatively steep learning curve for MRE and, therefore, radiologists and technologists must be trained in MRE techniques in order to capture high-quality images and interpret them accurately.^24^ Although rare, intravenous (IV) MRE contrast agents, if used, can pose risks for patients with allergies to the contrast material. In addition, patients with impaired renal function who have been exposed to gadolinium-based contrast agents during MRE have a rare but serious risk of developing nephrogenic systemic fibrosis.^24^

### MRE activity scores

There is no single guideline-approved score for MRE, although of the MRE indices currently available to evaluate CD activity, the MaRIA has the most extensively evaluated operational characteristics. It is a responsive score composed of mucosal and bowel wall features, including bowel edema, bowel wall thickness (BWT), presence of ulcers, and relative contrast enhancement.^26^^,^^27^ The MaRIA score has some limitations. Nonpathologic segments contribute to the global MaRIA score; the global score is underestimated in patients with resected segments.^26^ In addition, it is time-consuming to measure BWT and relative contrast enhancement segment by segment.

The limitations of the MaRIA score led to the development of other MRE-based indices. The sMaRIA index has been developed to allow easier and faster inflammation and severity assessment in CD while maintaining the high accuracy of the original MaRIA score for both diagnosis and therapeutic response.^26^ Further advantages are that the sMARIA score for nonpathologic segments is zero, and components can be evaluated without contrast. The disadvantage is the low responsiveness when BWT is reduced but still abnormal without impacting the other components.

Two further MRE scores have been developed. The first is the London index, which includes BWT and mural T2 signal (edema) as inflammatory parameters. The score was derived using histopathology disease activity assessment in the terminal ileum as the reference standard without further disease severity grading.^28^ The London Index is accurate for diagnosing disease activity; however, it may be less responsive for quantifying disease severity, which is essential for therapeutic response assessment. The second MRE score is the Clermont score (C-score), which was developed and validated in 2024 and replaced relative contrast enhancement with the apparent diffusion coefficient using diffusion-weighted imaging sequences.^29^ The modified C-score for assessing transmural remission, which includes BWT (vs normal), presence of any ulcer, and presence of enlarged lymph nodes, is another easy-to-use tool for assessing transmural remission (C-score < 0.5) and transmural response (defined as either at least 50% improvement of C-score in each active segment at baseline [TR50] or at least 25% improvement of C-score in each active segment at baseline [TR25]) in patients with CD.^30^ A disadvantage of the C-score includes that there are limited data on the reproducibility of apparent diffusion coefficient measurements across different MRI platforms.

### Intestinal ultrasound

Intestinal ultrasound is a specialized addendum and complementary to the more common routine transabdominal ultrasound.^31^ Intestinal ultrasound has emerged as a noninvasive, real-time cross-sectional imaging technique, highly accurate for detecting moderate-to-severe endoscopic inflammation. As a complementary technique to IC, it can aid in diagnosing IBD and monitoring disease activity and treatment response.^9^

Intestinal ultrasound can visualize the colon (with limited transabdominal rectal views), motility, and the majority of the small intestine (except deep pelvic and some proximal jejunum segment), with limited assessment of the stomach and duodenum is possible.^32^^,^^33^ Intestinal ultrasound enables the evaluation of transmural inflammation (eg, BWT and bowel wall stratification), intra- and extramural vascularization, transmural ulcers, and extramural findings such as mesenteric inflammation and function, including real-time small bowel motility.^32^^,^^34^^,^^35^ Intestinal ultrasound has been shown to accurately detect complications such as strictures, abscesses, and fistulae.^32^^,^^34^^,^^35^

In addition, IUS is helpful in patients unable to undergo MRE due to metal implants, or conditions such as claustrophobia, difficulty with breath holds, or inability to remain still.^36^ Other advantages of IUS are its point-of-care use by the treating physician which affords the opportunity for simultaneous patient education, potential cost avoidance due to a reduced need for MRE and colonoscopy procedures, and shorter waiting lists compared to MRE in some countries.^9^^,^^37^

### Comparison of MRE vs IUS

The METRIC trial (ISRCTN03982913) compared the diagnostic accuracy of MRE and IUS for the presence, extent, and activity of small bowel CD in newly diagnosed patients or patients with established disease and suspected relapse.^38^ The results showed that MRE and IUS were highly accurate for detecting small bowel CD, with MRE and IUS achieving sensitivities of 97% and 92%, respectively. Intestinal ultrasound was significantly more sensitive than MRE for detecting colonic disease in newly diagnosed patients (67% vs 47%). For both MRE and IUS, sensitivity for colonic disease was higher in patients with relapsed disease compared with the newly diagnosed group. Compared with an IC standard, MRE had a higher sensitivity for terminal ileal disease presence than IUS (97% vs 91%). Magnetic resonance enterography and IUS were deemed valid first-line investigations and viable alternatives to IC; however, MRE may be better for detecting the full extent of small bowel involvement, particularly at diagnosis. Although MRE is widely used and has similar efficacy to IUS in detecting CD, it requires an oral contrast agent, making it a less favorable option than IUS. This is reflected by more patients finding IUS the most acceptable modality.

The cost of IUS has been shown to be cheaper than MRE. A retrospective, single-center study in the UK (2018) predicted that IUS could have replaced a significant number of MREs (55%) and lower gastrointestinal endoscopies (28%) which would have translated to a predicted annual cost saving of almost £500 000.^39^ In addition, a clinical service evaluation of imaging pathways at a single National Health Service site in the UK (January 2021-March 2022) investigated whether IUS was quicker, more acceptable and cheaper than alternative monitoring for patients with CD.^40^ The estimated costs per patient in the IUS pathway was £79, and this cost increased to £375 per patient for the MRE pathway, indicating a significant cost reduction using IUS. This difference was partly due to fewer healthcare interactions (£2980 vs £6051). There were also significant reductions in the average time to referral provision of the report (29 days vs 72 days) and the time to starting a new treatment plan (46 days vs 91 days), with IUS vs MRE. The quicker turnaround time for IUS compared with MRE, along with cheaper costs and a reduced patient burden, could facilitate screening of patients in clinical trials.

### Reducing clinical trial screening failures

Pre-screening with segmental IUS BWT may reduce the high rates of screening failures currently seen with SES-CD in patients with moderate-to-severe CD.^41^ Pre-screening with IUS could be used to establish patients’ eligibility and reduce unnecessary clinical trial screening procedures in cases in which objective inflammation is absent; however, further validation of this tool is needed.^41^

### Need for validated disease activity score(s) and definitions of response

The CDAI and Harvey-Bradshaw Index are valuable for stratifying patients into mild, moderate, or severe clinical disease activity in clinical trials; however, they do not reflect overall disease activity burden, including bowel damage and quality of life.^42^ Due to its noninvasive nature and ability to detect recurrence,^33^ IUS could be used more regularly in clinical trials to enhance our understanding of the dynamic improvements in inflammation. However, an unmet need remains for a simple, validated IUS disease activity score.

### Definition of response using IUS

Challenges of IUS use include interpreting and grading lesion changes in response to treatment and defining remission after treatment. In addition, only the worst segment is followed for endpoint assessment, even if several segments are involved.^43^ An expert consensus by the International Bowel Ultrasound (IBUS) group recommends that treatment response should be identified by reductions in BWT in the small and/or large bowel.^43^ In addition, they recommend that the ideal assessment of IUS response within the first year of treatment initiation should be at baseline, Week 14 ± 2, and between Weeks 26 and 52.^43^ Although not recommended by the IBUS group, an additional early timepoint within 2-4 weeks for this noninvasive endpoint might increase our understanding of the role of IUS in early predicting longer-term outcomes. However, the IUS routine early performance would be limited by feasibility. Intestinal ultrasound should also be repeated if elevated fecal calprotectin, symptoms, or clinical suspicion of a flare occur.^43^  Table 2 shows the consensus recommendations for IUS treatment response in IBD and guidance for defining strictures on IUS.^32^^,^^43^

**Table 2.** Consensus recommendations for IUS treatment response in CD.

	Topic	Consensus
IBUS	Machine recommendations	Treatment response can be assessed by IUS
		Response should be assessed with the same type of probe (high-frequency vs abdominal probe) and constant machine settings (Doppler scale, presets, etc.)
	Response rate	Response rate detected by IUS is comparable with the rate of improvement in luminal inflammation, assessed by endoscopy and the rate of MRE improvement
		Response rate in IUS is dependent on class of drug (5-ASA vs steroid vs immunosuppressant vs biologics), disease duration (new-onset vs long-term established disease), and histological composition of a pathological segment (active inflammation only vs fibrotic only vs combined)
		Response rate, in general, is different for strictures than luminal disease, phlegmons than luminal disease, and abscesses than luminal disease
	Length of disease	Length should be reported using involved colonic segment(s) (sigmoid colon, descending colon, transverse colon, ascending colon, cecum)
		For the terminal ileum, the length should be reported as the distance from the ileocecal valve (if possible) or as proximal small bowel
	Measuring BWT	Response depends on baseline thickness and should be reported in absolute (mm) and relative (%) change from baseline. Continuous measurements (1 dp of precision) are preferred over categories and should be reported as a mean of 2 measures in cross-section and 2 measures in longitudinal orientation
	Defining the worst segment	The worst segment is defined by the most pathological BWT; however, if 2 segments have the same BWT, the order of secondary parameters for defining the worst segment should be the grading of CDS, bowel wall stratification, and then inflammatory mesenteric fat
	Disease activity scores	If a score is used, the score should summarize measures of all individual segments
		Treatment response could be a combined change in one or more activity parameters, specified as a point reduction from an activity score (present or in the future), BWT (continuous) and/or CDS (ordinal), and/or bowel wall stratification (ordinal), and/or inflammatory mesenteric fat
	Response definition and timing of assessment in CD	Identified by reduction of BWT (continuous measure >25% or >2.0 mm or >1.0 mm and 1 CDS reduction)
		IUS complications (strictures, phlegmons, abscesses) should be assessed for response
		Response should initially be assessed in the small and large bowel after treatment initiation (regardless of treatment) at 14 ± 2 weeks^a^
		Ideal assessment of IUS response within the first year of treatment initiation/escalation/change is at baseline, Week 14 ± 2, AND between 26–52 weeks + IUS depending on elevated fecal calprotectin OR symptoms OR clinical suspicion of flare
	Transmural remission, definition, and timing of assessment in CD	Transmural remission of the small and large bowel is defined by BWT ≤3 mm with normal/0 CDS
		In some patients, sigmoid colon may contain an enlarged muscularis propria (outer hypoechoic layer typical in diverticular disease), allowing for BWT up to 4 mm without resembling active inflammation
		Transmural remission should be assessed after treatment initiation (regardless of treatment) between 26 and 52 weeks
		Transmural remission may already occur at Week 12 but with increasing likelihood up to 1 year (maybe 2 years)
STAR consortium^b^	Improved features from successful anti-inflammatory treatment of small bowel CD strictures on IUS	Stricture length, BWT, luminal narrowing, pre-stenotic dilation, motility abnormalities, loss of bowel wall layer stratification, mesenteric inflammatory fat, penetrating disease, ulceration, mural or peri-enteric hyperemia, comb sign
	Improved features from successful anti-fibrotic treatment of small bowel CD strictures on IUS	Stricture length, BWT, luminal narrowing, pre-stenotic dilation, motility abnormalities
	Features of treatment failure or re-obstruction of small bowel CD strictures on IUS	Stricture length, BWT, luminal narrowing, pre-stenotic dilation, motility abnormalities, penetrating disease, ulceration, mural or peri-enteric hyperemia
	Treatment response for stricture length	Improvement of more than 25% in BWT
		Improvement of more than 50% in luminal narrowing
	Improvement in pre-stenotic dilation	Reduction in absolute diameter of more than 25%
		A bowel diameter of less than 2.5 cm
	Treatment response in strictures with a fixed narrowing (ie, a rigid segment of bowel with a narrowed lumen) without pre-stenotic dilation	Improvement in stricture length, BWT, luminal narrowing, motility abnormalities, loss of bowel wall layer stratification, ulceration, or mural or peri-enteric hyperemia
	Measurement of improvement in mural and peri-enteric hyperemia (using CDS)	A 1-point or 2-point reduction in the Limberg score and modified Limberg score
		A 1-point decrease with or without a BWT by more than 25% in the IBUS-SAS CDS subscore
	Assessment of treatment response on IUS as the primary outcome	Measure at 3 time points: Weeks 12, 24, and 52
	Type of imaging	Conventional IUS with Doppler imaging or IV contrast is appropriate to assess the inflammatory component of small bowel stricture
		Greyscale IUS with Doppler imaging is appropriate for assessing the fibrotic component of a small bowel stricture

a In a subset of patients, response after steroids or biologics may occur already after 4 weeks.

Early IUS assessment may, in certain situations, be beneficial between Weeks 4 and 8.

b Only statements deemed as appropriate have been included.

Abbreviations: 5-ASA, 5-aminosalicylate; BWT, bowel wall thickness; CD, Crohn’s disease; CDS, color Doppler signal; IBUS, International Bowel Ultrasound; IBUS-SAS, International Bowel Ultrasound Segmental Activity Score; IUS, intestinal ultrasound; IV, intravenous; MRE, magnetic resonance enterography; STAR, Stenosis Therapy and Anti-Fibrotic Research.

### IUS activity scores

Although multiple scores have been developed, there is no single, guideline-approved score for IUS, and a standard, widely validated score needs to be endorsed.^33^ Many IUS activity scores have been developed and validated using IC as a reference standard, incorporating parameters identified through multivariate analysis as independent disease activity and severity predictors. This approach aims to make assessments more systematic and reproducible, allowing for uniform reporting similar to standards currently available in endoscopy.^44^^,^^45^ These IUS activity scores focus on BWT, increased color Doppler signal (CDS), disrupted mural stratification, and inflammatory fat wrapping. Descriptions of the most promising IUS scores are shown in Table 3.^46–49^ Briefly, the simple ultrasound activity score for CD (SUS-CD) is 0–5 based on BWT and CDS and correlates well with SES-CD. It detects endoscopic activity accurately (NCT03481751).^49^ The Bowel Ultrasound Score (BUSS), which incorporates BWT and CDS, accurately detects endoscopic activity (SES-CD > 2) with a threshold >3.5 and predicts disease progression.^46^^,^^47^ A BUSS ≤ 3.5 after induction (Week 12) predicts long-term endoscopic remission. A persistent BUSS >3.5 predicts worse outcomes, including increased need for steroids, surgery, and hospitalization.^46^^,^^47^ The IBUS Segmental Activity Score (IBUS-SAS) has a more granular and responsive score of 0–100; however, the scoring system is also more complex (using all four components), limiting IBUS-SAS use as a point-of-care test. Nevertheless, IBUS-SAS may be useful in clinical trials and will likely better assess treatment response.^48^ Although these IUS activity scores are promising, external validation is required before use in clinical trials and clinical practice. So far, 2 studies have been conducted to validate the correlation between IUS scores and endoscopic activity in CD.^44^^,^^50^ A prospective study compared IBUS-SAS, BUSS, simple-US, and SUS-CD scores and reported that all IUS scores strongly correlated with endoscopic findings.^44^ However, the IBUS-SAS performed better in assessing severe endoscopic activity due to its more detailed scoring system, which can help to stratify different disease activity levels. The BUSS and SUS-CD scores were also compared in 284 participants enrolled in the METRIC (ISRCTN03982913) trial who underwent IUS and MRE, including those with terminal ileal biopsies.^50^ Both scores were clinically viable with reasonable sensitivity and specificity for terminal ileal CD, especially when compared with MRE as a reference standard. SUS-CD had a significantly higher sensitivity but a significantly lower specificity than BUSS.

**Table 3.** IUS scores: BUSS, SUS-CD, and IBUS-SAS.

Score	Description
BUSS	• Worst affected section = 0.75 × BWT (mm) + 1.65 × BWF • BWF defined as 0 (absence) or 1 (presence) of vascular signals at CDS • BUSS > 3.5 is an indicator of endoscopic activity (SES-CD > 2)
SUS-CD	• Worst affected section = BWT + CDS • Score ranges from 0 to 5 • Score 0 = BWT, <3 mm + CDS, no or single vessel per cm^2^ • Score 1 = BWT, 3–4.9 mm + CDS, 2–5 vessels per cm^2^ • Score 2 = BWT, 5–7.9 mm + CDS, >5 vessels per cm^2^ • Score 3 = BWT, ≥8 mm
IBUS-SAS	• Any segment = 4 × BWT (mm) + 15 × i-fat + 7 × CDS + 4 × BWS • CDS defined as 0 (normal), 1 (short signals), 2 (long signals inside bowel), 3 (long signals inside and outside bowel) • I-fat defined as 0 (normal), 1 (uncertain), 2 (present) • BWS defined as 0 (normal), 1 (uncertain), 2 (focal), or 3 (extensive [>3 cm]) • Score ranges from 0 to 100

Abbreviations: BUSS, Bowel Ultrasound Score; BWF, bowel wall flow; BWS, bowel wall stratification; BWT, bowel wall thickness; CDS, color Doppler score; IBUS-SAS, International Bowel Ultrasound Segmental Activity Score; i-fat, inflammatory fat; IUS, intestinal ultrasound; SUS-CD, Simple Ultrasound Activity Score for Crohn’s Disease.

Three ongoing trials that are attempting to validate IUS activity scores are the EXTENT (NCT06647823), USE-IT (NCT05407350), and ECHOCROHN (NCT03439826) trials. The EXTENT trial will assess and quantitatively measure structural bowel damage in CD with the IUS Lèmann Index.^51^ The primary objective is to demonstrate whether IUS can replace MRE and IC to evaluate small bowel and colonic global damage (excluding rectum) in patients with CD. The USE-IT trial aims to prospectively validate a novel IUS activity index for CD and correlate index treatment responsiveness with IC (SES-CD) and MRE (sMaRIA).^52^ Developing this novel IUS CD activity score may improve standardization and consistency in global IUS utilization, through broader adoption of reliable, accurate disease activity evaluation in clinical trials and real-world clinical practice. This may lead to accelerated uptake of treat-to-target strategies in patients with CD. Finally, the ECHOCROHN trial aims to investigate the interobserver ­reliability of an IUS score.^53^

## Cross-sectional imaging in current clinical trials

After the growing interest in achieving transmural remission, cross-sectional imaging techniques such as IUS and MRE, which allow the visualization of transmural and extramural inflammation, are increasingly used in clinical trials.^13^ Current trials investigating the use of MRE and IUS are shown in Tables S1 and S2 and summarized below.^52–65^

The STARDUST trial (ustekinumab; NCT03107793) was one of the first to incorporate centrally read IUS in a substudy to evaluate treatment response, including transmural remission in patients with moderate-to-severe CD.^66^ Four ultrasound components were used to define IUS response and transmural remission, including normalization of BWT (BWT < 3 mm), absence of CDS (no hyperemia), normalization of bowel wall stratification, and absence of mesenteric inflammatory fat. Agreement between IUS and endoscopy at baseline was >90% in defining the most affected bowel segment, including the terminal ileum (92%), overall bowel segment (80%), and specific colon segment (63%). The rate of transmural remission (normalization of 3 of 4 IUS parameters with the remaining not assessed/not assessable) with ustekinumab was 24% at Week 48. Intestinal ultrasound response (defined as a reduction of ≥25% from baseline in BWT) was observed at Week 4 (26%) and continued through Week 48 (46%). At Week 48, IUS response and transmural remission were higher in the colon than the terminal ileum (63% vs 40% and 50% vs 13%, respectively). The substudy suggested that IUS was complementary to endoscopy for assessing CD activity. Responses were observed early after treatment initiation and were more pronounced in colon than in the terminal ileum. This trial helped pave the way for IUS use in other clinical trials.

VECTORS (NCT06257706) is an ongoing, phase 4, randomized, controlled, parallel-group, open-label study to evaluate transmural remission as a treatment target in approximately 300 participants with moderately to severely active CD using IUS.^54^ The study aims to assess whether treating to a target of corticosteroid-free IUS outcomes + clinical symptoms + biomarkers is superior to a target of clinical symptoms + biomarkers alone in achieving corticosteroid-free endoscopic remission, measured by SES-CD. In group 1, participants will be treated over 48 weeks to achieve a target of corticosteroid-free, IUS-based outcomes + clinical remission + biomarker remission. At Weeks 22 and 30, the IUS-based target is IUS response, and at Week 38, the treatment target will be transmural remission. In group 2, participants will be treated over 48 weeks to achieve a target of corticosteroid-free clinical remission + biomarker remission. All participants will receive IV vedolizumab induction (300 mg) at Weeks 0, 2, 6, and 10, followed by IV vedolizumab maintenance treatment (300 mg every 8 weeks [Q8W]) starting from Week 14. Treatment may be modified at Weeks 22, 30, and/or 38 based on the results of the target assessment at each of these time points.

The SUNRISE trial (NCT05192863) is investigating the percentage of patients who achieve complete remission 12 months after IV vedolizumab initiation in adults with CD in the real-world setting. Complete remission is defined as corticosteroid-free clinical, biochemical, and transmural remission, with transmural remission measured using IUS and defined as BWT < 3 mm in all bowel segments. Furthermore, IUS is increasingly incorporated into clinical trials, with ongoing studies investigating if IUS can predict and monitor postoperative recurrence after ileocecal resection, monitor pediatric patients, assess treatment response, and explore management changes after IUS.

### IUS in pediatric patients

There is a paucity of clinical trial data on the use of IUS in pediatric patients.

A single-center, blinded, cross-sectional cohort study of 82 children and young adults (median age: 16.5 years) assessed the accuracy of segmental IUS parameters and scores to detect segmental SES-CD activity.^41^ Segmental IUS BWT was shown to be highly accurate to detect moderate-to-severe endoscopic inflammation and may be an ideal tool for prescreening patients to help reduce trial screening failures.

Intestinal ultrasound can be used for measuring BWT in children and adolescents. The Pediatric Crohn Disease Intestinal Ultrasound score was developed and shown to be an easy-to-use and reliable IUS score for measuring disease activity in pediatric patients with CD (median age: 15 years).^67^ BWT was categorized into low (1 point: 2–3 mm [terminal ileum] and 1.6–2 mm [colon]), medium (2 points: 3.0–3.7 mm [terminal ileum] and 2.0–2.7 mm [colon]), and high (3 points: >3.7 mm [terminal ileum] and >2.7 mm [colon]). Mesenteric fat (1 point) was also an independent predictor of disease activity in the colon, but not the terminal ileum. A score of 0 points had a high probability of no disease activity (sensitivity 82% [terminal ileum] and 85% [colon]), while a score of 3 points had a high probability of disease activity in both the terminal ileum and the colon (specificity, 88% and 92%, respectively).

A single-center, cross-sectional pilot study assessed the diagnostic performance of BWT measured using IUS compared with endoscopic disease activity in children suspected of having IBD. They found that increasing BWT was associated with increasing endoscopic activity in pediatric IBD and suggested that the optimal BWT cutoff value for detecting active disease may be less than that seen in adults.^68^ In a retrospective, cross-­sectional study of 98 pediatric patients (median age: 15.2 years) with IBD who underwent IUS after achieving sustained deep remission (defined as absence of ulcerations and SES-CD = 0 or MES = 0 on ileocolonocscopy and/or transmural healing on MRE and steroid-free clinical remission), the primary endpoint was median segmental BWT on IUS for sigmoid colon, descending colon, transverse colon, ascending colon, and terminal ileum.^69^ BWT on IUS in children with IBD in sustained remission was <3 mm, which is the consensus definition of transmural healing in adults and is unaffected by age, sex, and bowel segments.

Intestinal ultrasound can also be used for monitoring treatment response in children. A retrospective study showed that IUS-SAS at 22-38 weeks was an effective index for monitoring endoscopic response to infliximab therapy in 30 children with CD.^70^ In addition, a prospective longitudinal cohort study showed that early IUS response (Week 8) to biological therapy predicted endoscopic remission in 44 children with ileal CD.^71^ A total of 29 children achieved endoscopic remission (median age: 14 years), while 15 did not (median age: 13 years). Early reduction in BWT on IUS was highly predictive of treat-to-target endoscopic remission; a ≥ 18% decrease in BWT at Week 8 (end of biologic therapy induction) predicted treat-to-target endoscopic remission with the highest accuracy (sensitivity, 100%; specificity, 97%). Additionally, children with ileal CD with lower BWT at baseline are more likely to achieve endoscopic remission after treatment with biologic therapy, regardless of early IUS responsiveness. The authors suggested that directly monitoring change in BWT could be used in an IUS-dominant tight control strategy to guide therapeutic optimization with the potential to drive endoscopic remission rates higher in children with ileal CD than monitoring with traditional biomarkers.

### IUS at the bedside

There are several benefits to using IUS in clinical practice and clinical trials. As noted, IUS is inexpensive and quicker to complete than MRE, and unlike MRE, it generates the results in real-time.^40^^,^^45^ Intestinal ultrasound does not have the preprocedural requirements for IC or computed tomography/MRE imaging (eg, scheduling, fasting, bowel preparation, and oral contrast). Intestinal ultrasound, therefore, has the potential to reduce inefficiencies and nonadherence related to lack of compliance with preprocedural requirements.^36^ This is specifically important in patients who have comorbidities, with a higher risk of undergoing invasive or contrast procedures, who are pregnant, or who need frequent disease activity assessments. Intestinal ultrasound can be performed during an outpatient IBD consultation, eliminating the need for a separate appointment and minimizing recovery time and time away from work, school, or family. In addition, IUS does not require sedation, postoperative patient monitoring, or a specialized procedure room, allowing integration with existing appointments.^72^^,^^73^ Finally, given the visual disease demonstration, there is a significant potential for patient education and engagement in their treatment management. Intestinal ultrasound has increased patient’s disease understanding and treatment adherence.^74^^,^^75^ Considering these advantages, it is rather unsurprising that patients have reported a preference for IUS over MRE and colonoscopy.^76^

### Barriers to IUS use

Barriers to IUS use in both clinical trials and real-world practice exist and often overlap. Intestinal ultrasound is increasingly recognized as a valued tool for IBD disease monitoring and has been used for several decades for routine clinical care in Germany and Italy.^77^ It is becoming the standard of care throughout parts of Europe, Australia, Asia, and Canada^77^ and has been included in many IBD centers in the United States; however, IUS is not yet performed worldwide as standard of care.^78^ Barriers to widespread IUS use include a lack of adoption by leading centers, a lack of educational opportunities, and an unclear path to remuneration. Broader incorporation of IUS could bridge the existing gap between clinical trials and real-world practice.

To address the unmet need for standardization of image acquisition, an expert consensus panel by European Crohn’s and Colitis Organization and the European Society of Gastrointestinal and Abdominal Radiology has provided guidance on optimal reporting requirements for cross-sectional imaging in IBD, including IUS.^33^^,^^79^ Guidelines endorse using IUS as an appropriate first-line investigation and viable alternative to IC.^45^^,^^80^

Although central reading by experienced practitioners may minimize observer variation and bias and improve reliability in clinical trials, there remains the potential for inter-rater variability in operator-dependent image collection.^81^ Although a wide range of ultrasound machines exist,^82^ not all are suitable for high-quality acquisition.^83^ The IBUS consensus does not stipulate which type of machine should be used, but does state that it is essential to use the same probe type (mid/high-­frequency vs abdominal) with constant machine settings when measuring interval treatment response.^43^ To ensure consistency, the same machines fulfilling mandatory requirements might be used throughout all trial centers.

The process for uploading cine-loops on the central reading platform is time-consuming; data must be deidentified, downloaded from the machine, and then uploaded to the central reading platform.^66^ A potential solution to this would be to use artificial intelligence (AI) to deidentify data and upload it to central reading platforms, although this approach is still in development.^84^ Artificial intelligence applications in IUS could have a number of benefits, including improving the performance of inexperienced operators by facilitating the learning curve, allowing for quicker and better-quality examinations, reducing interobserver variability, and improving the overall accuracy of activity assessment.^85^ In addition, AI could provide an opportunity to better understand the underlying patterns and timing of response, through identification of new or under-recognized imaging features such as the quality of the mesenteric inflammation.

Another IUS limitation is a lack of local expert capacity, caused by several factors, including a lack of sufficient training of healthcare providers.^37^ Not all clinical trial centers have trained personnel who can perform both ICs and IUS; therefore, IUS adoption necessitates more trained personnel at each site.^9^ Most gastroenterologists and radiologists in the United States have not received IUS training. In contrast, in Italy and Germany, where IUS use has traditionally been more widespread, all doctors specializing in gastroenterology are trained in abdominal ultrasounds/IUS.^9^^,^^78^ In sites with no IUS expertise, colleagues may be reluctant to take time away from the department to attend training courses, may need additional planning to attend courses, as well as resources required to participate. A Delphi panel has released consensus recommendations highlighting the IUS training standards needed, including lists of knowledge, technical skills, and interpretation skills required for performing IUS.^86^ Obtaining the official certification for a physician interested in getting trained in performing IUS takes several weeks of hands-on training.^72^ There is currently a very high demand to undergo the IBUS training curriculum (www.ibus-group.org), leading to a long waiting list.^37^ This has led to educational redesigns with a modified training curriculum utilizing an introductory eLearning module, which can be booked directly from the website. Further redesigns are expected, including advanced eLearning courses. Although IBUS initially focused on training colleagues in academic centers, these were not necessarily the high-recruiting centers in clinical trials. Identifying and expediting training in high-­recruiting centers for clinical trials remains essential.

Another limitation of using IUS in clinical practice is the precise localization and extent of the disease. Visualization of the proximal jejunum and rectum may be challenging^33^^,^^34^; however, most patients with CD have ileal, colonic, or ileocolonic manifestations that can be well visualized.^87^ An add-on baseline MRE can overcome this, and IUS can be applied going forward with identical findings.^37^ Furthermore, very mild mucosal disease may not be correctly captured with IUS or MRE, and IC or capsule endoscopy is required to detect these cases.^72^ Since ultrasound exhibits attenuation, IUS may be suboptimal due to compromised visualization in some patients, more often in patients with obesity/high body mass index.^72^ Although IUS image quality may be limited in obese patients, no significant difference was reported in IUS findings by weight in study of patients with IBD, suggesting that obesity does not preclude image quality.^82^

Another unmet need is access to suitable equipment. Acquiring and maintaining high-quality ultrasound equipment can be expensive. Incorporating IUS into routine practice may require significant initial investment.^9^ A mid-range ($40,000–$50,000) to high-end ($80,000–$120,000) ultrasound machine is recommended, and a mid- to high-frequency probe (incorporating frequencies of 5–8 MHz or higher) is necessary for examining the intestines. A low-frequency (1–6 MHz) convex probe may be used for a broader and deeper view of the abdomen, to map the bowel and complement the high-resolution detail of the intestinal wall.^9^ These costs may limit implementation in smaller and less dedicated centers.

The IUS procedure duration may vary based on the operator’s experience, patient characteristics (eg, the presence of complications that require careful measurement of the details), and the number of ultrasound machines available. This requires cautious scheduling consideration, at least during the initial adoption of IUS in a specific center.^72^ In addition, it is highly recommended that images be archived in a picture archiving and communication system, allowing other healthcare professionals to make easy comparisons at the patient’s follow-up visits.

There remains an unmet need for developing a reimbursement model in a number of countries to ensure the sustainability of service. Physician remuneration or compensation for time is variable within countries and regions. In many parts of the world where IUS is not part of the standard of care, billing codes and procedure codes are available for a limited abdominal ultrasound (Current Procedural Terminology [CPT] 76705); however, there is an unmet need for a specific CPT code for IUS. This needs to be developed once the time required to perform and interpret the IUS examination is better understood.^9^ Reimbursement is expected to depend on the site of service, and obtaining IUS privileges should follow local hospital system guidelines.^9^

### Ultrasonography techniques in development

Two other ultrasonography techniques are available: small intestine contrast-enhanced ultrasonography (SICUS) and ­contrast-enhanced ultrasound (CEUS). In SICUS, oral contrast medium is ingested before performing IUS to allow improved bowel wall visualization and increased separation between adjacent bowel loops.^45^ This may have some benefit over conventional IUS; however, it is more laborious, time-consuming, and less acceptable to patients, hampering its widespread adoption.^45^ In CEUS, IV contrast is administered simultaneously with performing IUS. This allows differentiation between an inflammatory mass and abscess, and several trials, including ongoing trials, examine its utility in differentiating between inflammation and fibrosis.^45^^,^^88–91^ Unfortunately, there is a significant lack of standardization and associated cost, limiting widespread implementation of this technique.

## Conclusions

Intestinal ultrasound is increasingly being investigated in clinical trials to assess transmural inflammatory changes in CD, and IUS transmural remission or response may become an accepted endpoint. Intestinal ultrasound has several advantages, including being a noninvasive technique that does not require sedation, bowel preparation, or exposure to ionizing radiation. It allows assessment of disease activity in real-time, making it a valuable tool for repeated monitoring of treatment response and disease progression. It has high sensitivity and specificity for detecting inflammation and complications such as strictures, abscesses, and fistulas. Patients also prefer IUS over other diagnostic modalities like IC due to its convenience and comfort. Compared with other imaging modalities like MRE and CT, IUS is relatively cost-effective and can be performed at the point-of-care. However, the effective use of IUS requires specialized training for healthcare providers to ensure accurate acquisition and image interpretation. There remains an unmet need for standardized protocols and reporting systems to ensure consistency in using and interpreting IUS across clinical settings.

## Supplementary Material

izaf196_Supplementary_Data

## Data Sharing

Data sharing is not applicable to this article as no new data were created or analyzed.
