# Supplementary material for: Integrating Intestinal Ultrasound to Clinical Trials in Patients With Crohn’s Disease: Opportunities and Challenges
Source: Inflamm Bowel Dis. 2025 Sep 4;31(12):3429–42. doi: 10.1093/ibd/izaf196 (PMC12688078; doi:10.1093/ibd/izaf196)
Supplement: izaf196_Supplementary_Data [file izaf196_supplementary_data.zip › SUPPLEMENTAL APPENDIX.docx]

**SUPPLEMENTAL APPENDIX**

**Supplemental Table S1.** Clinical trials using MRE.

| **Trial name**  **Trial number (Status)** | **Study description** | **Central reading** | **Patients** | **Primary outcome** | **Key secondary outcomes** |
| --- | --- | --- | --- | --- | --- |
| Helmsley 3.0  NCT06533228  (Ongoing) | Abbreviated MRE | NR | Aged ≥10 with CD, undergoing MRE | Compare clinical MRE with abbreviated MRE (scan time 15 minutes, replace oral contrast with water) | Patient/carer preference |
| TRENCH-1  NCT05903066  (Ongoing; also called GET-AID) | Evaluation of transmural healing in CD | No | Adults with CD and >6 months of histological confirmation, indication for MRE | Objective definition of depth or grade of transmural healing in relation to radiologic signs observed during MRE | Inter- and intra-observer variability in radiological findings and transmural healing definitions |
| CROCO  NCT05420233  (Recruiting) | Characterization of CD progression using the Lémann Index | NR | Adults with newly diagnosed CD | Lémann Index over 1 year | Lémann Index over 2 and 5 years |
| METEOR  NCT06124287  (Recruiting) | Prediction of relapse after biologic discontinuation | NR | Luminal small bowel or colonic CD in clinical remission and biologic therapy to be stopped due to clinical remission only | To develop and evaluate a multivariable prediction model for early (within 1 year) disease relapse in patients stopping biological therapy, combining pre-existing clinical parameters with MRE parameters and compare with baseline model incorporating clinical variables alone | Establish which individual MRE finding/combination of findings best predicts disease relapse after stopping biologic therapy, including complete transmural healing |
| MANTRA  NCT06800326  (Recruiting) | Multicenter, non-randomized, prospective cohort study | NR | Patients aged ≥16 years with small bowel or colonic CD | Accuracy of MRE and IUS for classifying treatment response compared with a multidisciplinary consensus panel reference standard | NR |
| WAM  NCT06690632  (Not yet recruiting) | Compare water and polyethylene glycol preparation as oral contrast media in MRE | NR | Adults with CD and a need for MRE | Proportion of patients with an overall small bowel distension over 2 on a validated scale (0=poor to 4=excellent) | Tolerability |
| NCT03096379  (Completed) | Differentiating gut tuberculosis from CD using MRE | No | Adults with new-onset lower gastrointestinal symptoms of <3 months and Ileocecal mucosal lesions of uncertain diagnosis as evidenced by the presence of inflammation, ulceration, strictures, or nodules on colonoscopy | Small/large bowel transmural enhancement using MRE | Skip lesions in small/large bowel, vascular engorgement, mesenteric combing |
| NCT03541733  (Completed) | Bowel preparation for MRE | NR | Patients aged ≥14 years with CD and need for MRE examination and mid-gut tubing | Grade of bowel distension, degree of discomfort at 1 year | Accuracy of lesion detection, confirmed by endoscopy, at 1 year |
| NCT01593462  (Completed) | Comparative effectiveness of MRE, enteric US and US elastography imaging | NR | Children with small bowel CD and no prior treatment | Accuracy of imaging techniques | Costs and patient preference |
| NCT04321941  (Completed) | Phase 2, open-label, non-inferiority trial comparing CTE with MRE | No | Adults with CD and clinical indication for MRE of the small bowel | Radiological CD Activity score based on 18 morphologic entities and dynamic signs in the small bowel and colon | NR |
| NCT05203926  (Completed) | Pilot study to characterize findings of possible sacroilitis in CD | NR | Adults with CD and evidence of sacroiliitis on earlier standard of care MRE and known HLA-B27 status | Significance of sacroiliitis features/lesions seen on MRE | Measurement of Ankylosing Spondylitis Disease Activity, Bath Ankylosing Spondylitis Disease Activity Index, Bath Ankylosing Spondylitis Functional Index, Harvey-Bradshaw Index, and CD Activity Index |
| METRIC ISRCTN03982913  (Completed) | Comparative accuracy or MRE and IUS for assessing disease extent and activity | No | Patients aged ≥16 years with newly diagnosed CD or established CD and suspected relapse | Difference in per-patient sensitivity for small bowel disease extent (correct identification and segmental localization) for MRE and IUS against a construct reference standard (panel diagnosis) | Specificity, safety |
| CREOLE  NCT01183403  (Completed) | CEUS vs MRE to predict efficacy of anti-TNF therapy in CD | NR | Adults with CD, small bowel stricture, obstructive symptoms ≥3 (scale 0–6) over the previous 8 weeks, corticosteroid/ immunosuppressant treatment failure, and an indication for anti-TNF therapy | Percentage of failure defined by the development of at least 1 event from baseline to 6 months | Change in obstructive symptom scale over 6 months |
| NCT03646708  (unknown status) | Response assessment in small bowel CD using MRE and proteomic biomarkers | NR | Adults with confirmed small bowel CD with active disease who initiated a biologic | Corticosteroid-free remission at 1 year | Mucosal healing in the terminal ileum (SES-CD) |
| ANDI  NCT03134586  (Completed) | Prospective, blinded, multicenter study of pillcan colon capsule endoscopy, MRE and US in patients with suspected CD | No | Patients aged ≥15 years with clinical suspicion of CD, negative serologic markers for celiac disease, negative stool culture for pathogenic bacteria, negative microscopy for intestinal parasites and fecal calprotectin >50 mg/kg | Sensitivity and specificity of pillcan colon capsule endoscopy, MRE and US for diagnosis of CD located in the terminal ileum and colon; correlation of disease severity and interobserver agreement between techniques | Diagnostic yield of proximal CD, patient-reported experience |
| ProSpA-CD  NCT03817983  (Completed) | MRE as screening tool for axial spondylarthritis in CD | NR | Adults with CD who have had an MRE scan | Determine the validity, sensitivity and specificity of MRE as a screening tool for axial spondylarthritis in patients with CD using dedicated axial MRI scans with clinical assessment as the gold standard | Devise an algorithm of clinical indices that can be used as a screening tool for axial spondylarthritis in patients with CD |

CD, Crohn’s disease; CEUS, contrast-enhanced ultrasound; CTE, computed tomography enterography; IUS, intestinal ultrasound; MRE, magnetic resonance enterography; MRI, magnetic resonance imaging; NR, not reported; US, ultrasonography; SES-CD, Simple Endoscopic Score for Crohn's Disease; TNF, tumor necrosis factor.

**Supplemental Table S2.** Clinical trials using IUS.

| **Trial name**  **Trial number (Status)** | **Study description** | **Central reading** | **Patients** | **Primary outcome** | **Key secondary outcomes** |
| --- | --- | --- | --- | --- | --- |
| VECTORS NCT06257706  (Ongoing)^54^ | Phase 4, randomized, controlled, parallel-group, open-label | IUS, endoscopy and histology assessments will be centrally read | 304 participants with moderately to severely active CD  Treatment targets: Treating to target of CS-free IUS outcomes + clinical symptoms + biomarkers vs clinical symptoms + biomarkers alone for endoscopic remission | Percentage of participants with CS-free endoscopic remission in groups 1 and 2 at W48 | Percentage of participants in groups 1 and 2 at W48 with:   - CS-free endoscopic remission + endoscopic remission + clinical remission - CS-free IUS response + endoscopic remission + clinical remission - CS-free endoscopic remission + clinical remission - CS-free endoscopic response + clinical response - CS-free endoscopic response - Endoscopic remission - IUS response - Histologic remission - Histologic response   Percentage of participants in groups 1 and 2 at W14, 22, and 48   - CS-free clinical remission - CS-free clinical response   Percentage of participants in group 1 at W14, 22, 30, 38, and 48   - Endoscopic remission - IUS response   Percentage of participants with biomarker response in groups 1 and 2 at W48, 64, 80, and 96  Percentage of participants in groups 1 and 2 at W6, 14, 22, 30, 38, 48, 64, 80, and 96 with   - CRP response - Fecal calprotectin response   CDAI total score and corresponding change from baseline (W6, 14, 22, 30, 38, 48, 64, 80, and 96)  SES-CD total score and corresponding change from baseline at W48  BWT measured by IUS in mm and corresponding change from baseline at W14, 22, 30, 38, and 48  CDS and corresponding change from baseline at W14, 22, 30, 38, and 48  IBUS-SAS (per segment and total score) and corresponding change from baseline at W14, 22, 30, 38, and 48  HRQoL measured by: PRO-2 score, SIQ-CD, IBDQ score, and Urgency NRS  Time to CD-related complication from randomization through W96 in both groups, and time to each component of CD-related complications  Percentage of participants who switched to an alternate biologic  AEs, SAEs, and AESIs |
| NCT02488005  (Completed)^55^ | Interventional diagnostic study using IUS | NR | 15 infliximab-naïve patients aged 6 to 21 years who were initiated on infliximab | Change in BWT at W14 + change in weighted Pediatric CDAI from baseline to W14 | Changes in fecal calprotectin and erythrocyte sedimentation rate from baseline to W14; CRP, infliximab level, and anti-infliximab antibodies at W4 |
| NCT02330458  (Unknown)^55^ | Prospective, observational study | NR | 20 patients aged 6–23 years with CD involving the distal small bowel and/or proximal colon initiating infliximab treatment | Change in BWT on small bowel ultrasound at W54, association between BWT on small bowel ultrasound change in BWT from W0 to W14, and the presence of remission at W54 | Change in fecal calprotectin from baseline to W54 |
| SUNRISE NCT05192863  (Active, not recruiting)^56^ | Non-interventional, prospective study | NR | Adults initiating IV vedolizumab induction treatment for CD | Complete remission 12 months after initiation of vedolizumab (measured by CS-free clinical, biochemical and transmural remission, with transmural remission defined as BWT <3 mm for all bowel segments in IUS) | Percentage of participants who achieve complete remission up to Month 18, percentage of participants who achieve the individual components of the primary outcome up to Month 1, percentage of participants with change (and reason for change) in vedolizumab dosing frequency, percentage of participants with acceptance of IUS monitoring and assessment as measured on a 6-point visual analog scale, percentage of patients with improved HRQoL as measured by SIBDQ, safety, and HCRU |
| VEDIAN NCT06180382  (Not yet recruiting)^57^ | Phase 4, randomized, parallel assignment comparison of vedolizumab and adalimumab dose intensification in patients with loss of response or biomarker activity to first-line adalimumab | NR | 220 adult patients with CD with a secondary loss of response to adalimumab | Clinical and biomarker remission (composite score) at W24 | Deep remission (primary outcome + mucosal remission and no treatment failure) by W24; clinical remission, fecal calprotectin, CRP, endoscopic remission as measured by different methods including magnetic resonance imaging, number of ulcerations, Lewis score, CDEIS, and BWT at W24; treatment failure, AEs, changes in IBDQ-32 score, clinical and biomarker remission, mucosal remission, and clinical decision support tool score |
| USE-IT NCT05407350 (Recruiting)^52^ | Validation of an IUS activity index over 50 weeks | Central reading of all imaging data | 110 adult patients with moderately to severely active CD and planned introduction of biologic therapy as per their treating gastroenterologist | The longitudinal and construct validity and treatment responsiveness of an IUS CDAI | The correlation of IUS CDAI and component items with IC, MRE, biomarkers, clinical activity scores, histology, HRQoL as measured by SIBDQ and PRO-2; the proportion of patients with IUS response and transmural remission after initiation of a biologic therapy approved for CD; the longitudinal validity of changes in IUS CD activity and component items after treatment with changes in IC, MRE, biomarkers, clinical disease activity scores, HRQoL, PRO-2, and histology; the correlation of terminal ileal small bowel peristalsis with IUS CDAI and component items, MRE, and IC with CD activity; the correlation of IUS CDAI and component items with patient outcomes, including ED visitation, hospitalization, and surgery |
| INSIGHT NCT05713409 (Recruiting)^59^ | Prediction and monitoring of postoperative recurrence by using IUS after ileocecal resection | NR | 120 patients aged ≥16 years with CD who are undergoing ICR or re-resection, with IUS performed at 3, 6, 12, 18, 24, 30, and 36 months | To investigate if IUS (measured by brightness mode [B-mode] and CDS) in combination with fecal calprotectin at 3 months after ICR is an early surrogate marker of endoscopic disease recurrence (defined by a Rutgeerts score ≥i2) at 6 months | To assess if IUS alone at 3 months after ICR is an early surrogate marker of endoscopic disease recurrence at 6 months; to assess if IUS alone or in combination with fecal calprotectin at 6 months after ICR is accurate in detecting endoscopic recurrence at 6 months (as defined by a Rutgeerts score ≥i2 and SES-CD ˃3); to develop an IUS score for postoperative recurrence in CD; to assess if IUS alone or in combination with fecal calprotectin at 12 months after ICR is a surrogate marker of endoscopic disease recurrence at 18 months and/or clinical outcomes at 18, 24, 30, and 36 months; to compare IUS and the Rutgeerts score upon endoscopy at 6 months after ICR for their prognostic role on clinical outcomes upon 3 years of follow-up; to assess if SWE during IUS at 3, 6, and 12 months is predictive of anastomotic stenosis upon follow-up; to assess the feasibility of IUS in the postoperative settings for different types of anastomosis; to assess the diagnostic accuracy of fecal calprotectin at 3 and 6 months to predict and determine endoscopic disease recurrence, respectively |
| NCT03435016  (Completed)^60^ | Comparative study of MRI, IUS, and capsule endoscopy for assessing treatment response | No | Adult patients with endoscopically active CD (SES-CD ≥3), clinically active CD (HBI ≥150, and a clinical indication for medical treatment with CS or biologics | Sensitivity and specificity of small bowel colon capsule endoscopy, MRE, and IUS for the diagnosis of ulcer healing in the terminal ileum and colon compared with IC | Diagnostic accuracy of fecal calprotectin and CRP; changes in bowel wall thickening, SWE, and Limberg score under medical treatment assessed with IUS |
| NCT05636657  (Not yet recruiting)^61^ | Intestinal color ultrasound (by SUS-CD) compared with capsule endoscopy (by CECDAI) for monitoring CD | NR | 50 patients with CD and active lesions as measured by color ultrasound or capsule endoscopy at the time of inclusion | SUS-CD and CECDAI at baseline and Month 3 of the treatment |  |
| ECHOCROHN NCT03439826  (Completed)^53^ | Validation and interobserver reproducibility of an IUS score | NR | Adult patients with CD benefiting from abdominal ultrasound as a part of usual follow-up | Interobserver reproducibility (junior/senior) of an ultrasound score of CD activity |  |
| NIMBUS NCT05673278 (Recruiting)^62^ | A 2-year feasibility study of IUS monitoring of pediatric IBD | No | Patients aged 2–19 years with IBD who are visiting hospital for clinical appointments (e.g., clinic, infusions, endoscopy) or inpatients in hospital with flare | Feasibility metrics (recruitment rate, retention/follow-up rate, availability rate of ultrasound scan attendance and specific parameters), and ultrasound parameter score total (composite measure of BWT, CDS, loss of wall layer stratification, loss of haustration, fatty wrapping, motility in thermal index, lymphadenopathy, and access/stricture) | Increase or decrease in medical management up to 12 months post IUS; complications (surgeries, admissions, flare of symptoms) up to 12 months post IUS |
| REASON NCT06408935 (Recruiting)^58^ | Phase 3b, open-label, multicenter study of guselkumab | NR | Adults with clinically active CD, active transmural activity in at least 1 segment, and inadequate response/intolerance to conventional therapy and primary non-response or secondary loss of response, or intolerance to a maximum of 1 class of approved advanced therapies for CD | Percentage of patients with MaRIA <11 in all intestinal segments at W48 | Percentage of patients achieving MaRIA <11 in all intestinal segments at W16 and 96; percentage of patients achieving MaRIA <11 and a reduction of ≥5 points from baseline in all segments at W16, 48, and 96; percentage of patients achieving MaRIA <11 and MaRIA <7 in all segments and endoscopic remission (SES-CD ≤4 with ≥2-point reduction from baseline and no subscore >1) at W48 and 96; percentage of patients achieving MaRIA <11 in all segments, PRO-2 remission, and no worsening of abdominal pain or stool frequency from baseline at W16, 48, and 96; percentage of patients achieving MaRIA <11 in all segments and biomarkers remission at W16, 48, and 96; percentage of patients achieving MaRIA <11 in all segments, PRO-2, and endoscopic remission at W48 and 96; percentage of patients with MaRIA <7 in all intestinal segments at W16, 48, and 96; absolute value of global simple MaRIA score through W96; change from baseline in global simple MaRIA score through W96; percentage of patients achieving MaRIA <7 in all intestinal segments not receiving CS at W16, 48, and 96; percentage of patients achieving transmural segmental and transmural total response with IUS at W4, 8, 16, 48, and 96; percentage of patients achieving transmural remission with IUS at W4, 8, 16, 48, and 96; absolute value of IBUS-SAS through W96; change from baseline in IBUS-SAS through W96; percentage of patients achieving IBUS-SAS response at W4, 8, 16, 48, and 96; percentage of patients achieving BWT ≤3 mm for ileum and colon plus CDS 0, in all segments and participants not receiving CS at W4, 8, 16, 48, and 96; absolute value of and change from baseline in BWT through W96; absolute value of and change in SUS-CD Score through W96 |
| NCT03481751  (unknown)^63^ | Validation and interobserver reliability of a SUS-CD | No | Adults with CD scheduled for IC | To validate the SUS-CD scoring system using a validated endoscopic score (SES-CD) as reference standard and further to assess interobserver variability; conventional ultrasonographic parameters in SUS-CD: BWT, stratification, CDS, fatty wrapping | To assess if changes in the SUS-CD score correlate to clinical and biochemical disease activity parameters used in CD, and to examine if SUS-CD could be used to predict treatment effect |
| IUSS Crohn’s feasibility study NCT03939117  (Completed)^64^ | Prospective trial | No | Adult patients with CD scheduled to undergo elective surgery to remove part of the terminal ileum that is affected by CD | Number of intraoperative ultrasound assessments completed; number of AEs reported during surgery; incidence of CD identified at macroscopic intraoperative assessment of the small bowel by the surgeon, and intraoperative ultrasound assessment of the small bowel; the length of areas affected by CD identified at macroscopic intraoperative assessment of the small bowel by the surgeon; to compare the incidence of CD and length of areas affected by CD identified macroscopically by the surgeon with the intraoperative ultrasound assessment; to evaluate the feasibility of study delivery at 6-week follow-up; to record surgical and patient outcomes up to 6 weeks; to evaluate the direct and indirect costs of a standardized intraoperative ultrasound protocol |  |
| IUS-TTT study NCT06534216  (Recruiting)^65^ | Prospective observational pilot study | NR | Adult patients with active IBD | Change in management after IUS compared with standard of care at different time points (3, 6, and 12 months) | Prediction of clinical and biomarker responses at 3 and 6 months; prediction of transmural responses at 3, 6, and 12 months; prediction of transmural remission, and endoscopic response and remission at 12 months |

AE, adverse event; AESI, adverse event of special interest; BWT, bowel wall thickness; CD, Crohn’s disease; CDAI, Crohn’s Disease Activity Index; CDEIS, Crohn's Disease Endoscopic Index of Severity; CECDAI, Capsule Endoscopy Crohn’s Disease Activity Index; CDS, color Doppler signal; CRP, C-reactive protein; CS, corticosteroid; ED, emergency department; HBI, Harvey-Bradshaw Index; HCRU, healthcare resource utilization; HRQoL, health-related quality of life; IBD, inflammatory bowel disease; IBDQ, Inflammatory Bowel Disease Questionnaire; IBUS-SAS, International Bowel Ultrasound Segmental Activity Score; IC, ileocolonoscopy; ICR, ileocecal resection; IUS, intestinal ultrasound; IV, intravenous; MaRIA, magnetic resonance index of activity; MRE, magnetic resonance enterography; MRI, magnetic resonance imaging; NR, not reported; NRS, Numerical Rating Score; PRO-2, patient-reported outcome 2; SAE, serious adverse event; SES-CD, Simple Endoscopic Score for Crohn's Disease; SIQ-CD, Symptoms and Impacts Questionnaire for CD; SIBDQ, Short Inflammatory Bowel Disease Questionnaire; SUS-CD, Simple Ultrasound Score in Crohn’s Disease; SWE, shear-wave elastography; W, Week.
